# A Novel Computer Vision Model for Medicinal Plant Identification Using Log-Gabor Filters and Deep Learning Algorithms

**DOI:** 10.1155/2022/1189509

**Published:** 2022-09-27

**Authors:** Stephen Opoku Oppong, Frimpong Twum, James Ben Hayfron-Acquah, Yaw Marfo Missah

**Affiliations:** ^1^Department of ICT Education, University of Education, Winneba, Ghana; ^2^Department of Computer Science, Kwame Nkrumah University of Science and Technology, Kumasi, Ghana

## Abstract

Computer vision is the science that enables computers and machines to see and perceive image content on a semantic level. It combines concepts, techniques, and ideas from various fields such as digital image processing, pattern matching, artificial intelligence, and computer graphics. A computer vision system is designed to model the human visual system on a functional basis as closely as possible. Deep learning and Convolutional Neural Networks (CNNs) in particular which are biologically inspired have significantly contributed to computer vision studies. This research develops a computer vision system that uses CNNs and handcrafted filters from Log-Gabor filters to identify medicinal plants based on their leaf textural features in an ensemble manner. The system was tested on a dataset developed from the Centre of Plant Medicine Research, Ghana (MyDataset) consisting of forty-nine (49) plant species. Using the concept of transfer learning, ten pretrained networks including Alexnet, GoogLeNet, DenseNet201, Inceptionv3, Mobilenetv2, Restnet18, Resnet50, Resnet101, vgg16, and vgg19 were used as feature extractors. The DenseNet201 architecture resulted with the best outcome of 87% accuracy and GoogLeNet with 79% preforming the worse averaged across six supervised learning algorithms. The proposed model (OTAMNet), created by fusing a Log-Gabor layer into the transition layers of the DenseNet201 architecture achieved 98% accuracy when tested on MyDataset. OTAMNet was tested on other benchmark datasets; Flavia, Swedish Leaf, MD2020, and the Folio dataset. The Flavia dataset achieved 99%, Swedish Leaf 100%, MD2020 99%, and the Folio dataset 97%. A false-positive rate of less than 0.1% was achieved in all cases.

## 1. Introduction

Computer vision is a broad term that describes the computer performing the function of an eye by using different mathematical algorithms on a digital image. This area is concerned with the automated processing of images from the real world, extracting features, and interpreting information in real-time based on the user's requirements [1, 2]. The fundamental task in computer vision is image recognition [3]. Human vision is unique and superior in that it detects and discriminates against the objects around it with ease. It can perceive 3-D structures with perfection and also categorize them efficiently [4]. Computer vision is modelled after the human visual system which nonetheless is superior in detection, identification, and discrimination objects. With human vision being such a complex mechanism, computer vision can be thought of as an approximation of it [5].

Plant taxonomy is the science that aims in detecting, recognizing, describing, characterizing, and naming plants. Chemotaxonomic, anatomical, and morphological classifications are some of the techniques adopted for this science [6]. In comparison to chemotaxonomy, morphological and anatomical classifications are viewed as more traditional [7]. The key aspects that play vital roles in plant taxonomy are; plant identification which deals with the determination of an unknown plant in relation to a previously collected specimen and plant classification which places a known plant in a category based on its shared characteristics with other plants.

Despite the fact that the tropical vegetation of Ghana is abundant in medicinal plants, a thorough understanding of their spread and usage appears to be limited to the aged and herbalists [8, 9]. The majority of them learned about the plants through oral transmission or by employing them in traditional medicinal preparations. In general, information obtained on the subject of diversity and value of medicinal plants in Ghana, and their use, is made available through ethnobotanical means by a few individual researchers [10]. Herbal medicine is gradually becoming more widely accepted and used globally. This realisation is similar in Africa, where over 60% of its populace relies only on these plants for their primary healthcare needs, especially in underdeveloped nations [11, 12]. As a result, plants constitute a significant contributor to natural products and a vital component of health care. Traditional medicines are extremely important to the pharmaceutical business; in fact, traditional medicines account for a quarter of all prescribed pharmaceuticals worldwide. In comparison to synthetic medications, medicinal plants are chosen since they have fewer side effects and are more affordable [13]. Humans benefit from classifying medicinal plants in a variety of ways, thus it is critical to address this issue [14].

Attributes such as leaves, fruits, flowers, or the entire plant are mostly used to identify a plant. The use of the leaves is one most promising and reliable methods of identifying medicinal plants out of all the keys of identification. [15]. The use of plants as medicine has necessitated plant identification in order to identify whether or not a given plant has medicinal characteristics. When the untrained eye examines two plants closely, it is simple to confuse them. This makes plant identification a very important part of natural products and medicine that should not be overlooked since misidentification might have catastrophic effects. [10]. Plants go through several growth phases in different seasons and may have different shape characteristics as a result of environmental factors including climate change, topographical position, and so on. [16]. Furthermore, knowledge of plant species is vital for biodiversity protection. Using conventional keys to identify plants is complex, time-consuming and it is laborious for nonbotanists and provides a formidable obstacle for freshmen interested in obtaining specific expertise because it necessitates the use of scientific nomenclature. [17]. It is indeed difficult to discern between plants using their many morphological traits. High intraclass variability and small interclass variances are the key obstacles. [18]. Plant categories are tightly related, and some of their structural parts are closely related, resulting in low interclass differences. Furthermore, plants vary greatly in terms of size, colour, shape, and texture, and their appearance changes throughout the year, resulting in significant intraclass variance [19]. This study seeks to identify a plant using a proposed deep learning algorithm to perform the matching process that maps a leaf image to a plant category.

The rest of the study is outlined as follows: Section 2 is made of the literature review of the main concepts related to the study, Section 3 presents the methodology of the study which includes the feature extraction methods and the proposed model as well as the performance metrics, Section 4 analyses and discusses the results and the last section presents the conclusion and future works.

## 2. Literature Review

### 2.1. Deep Learning

Deep learning, a branch of machine learning, uses varied layers of algorithms (ANNs, or artificial neural networks) to model high-level abstractions with complex structures in data [2], and it is also based on data learning representations [20]. For each layer, a different interpretation of data that has been fed into them is provided [21]. Deep learning, inspired by the human brain information processing patterns, utilizes huge data in order for a given input to be mapped to specific labels. Convolutional neural networks, deep belief networks, deep neural networks, and recurrent neural networks are all deep learning architecture. Deep learning has been applied in various areas including natural language processing, audio and video recognition, computer vision, and automatic speech recognition and have produced tremendous results [22, 23].

Traditional machine learning algorithms consist of various stages which are preprocessing, feature extraction, feature selection, and classification. Feature selection stage plays a great role in these types of algorithms and might lead to incorrect classifications of classes if it is biased. Deep learning, however, overcomes this problem by automating the learning of features that are rich and complex [24]. Deep learning combines both feature extraction and classification at a go. With the emergence of big data, the concept of deep learning has expanded greatly [25].

Deep learning algorithms provide some level of abstraction of data and this is achieved with the number of its layers and their sizes [26]. In the general interpretation of deep neural networks, universal approximation theorem and probabilistic inference are used [27]. Deep learning has the following advantages; universal learning approach [28], robustness [29], generalization [30], and scalability [31]. Some drawback concept of deep learning include; a large data is required; data modules are complex; it is very expensive to train and it requires a classifier for comprehending mere learning results [32].

In machine learning, overfitting is one common problem, especially in deep learning, when training a model due to the large volume of parameters needed to train this kind of complex model. This occurs when the trained model does not generalize or predict well to unknown test data [33]. One way of dealing with overfitting is regularization which allows the model to deduce better to unknown data when training on a finite training set, or with an imperfect optimization procedure [34]. DropConnect, dropout, data augmentation, stochastic pooling, batch normalization, weight decay, early stopping, and ℓ1 and ℓ2 regularization are some of thecommon regularization strategies used to prevent overfitting. [35].

Optimizers are used to minimize a loss function or to increase the production efficiency. They are dependent on the model's learnable parameters i.e., biases and weights [36]. Popular optimizers include Adam (Adaptive Moment Estimation), RMSProp (Root Mean Square Propagation), Stochastic Gradient Descent (SGD), AdaGrad (Adaptive Gradient Descent), Momentum, and Adadelta [37]. The choice of the best optimizer relies, among others, on training data and a trade-off between speed and performance, the application, and network architecture [38]. Adaptive gradient algorithms in particular RMSprop and Adam, in modern machine learning models training, have demonstrated greater performance, e.g., deep neural networks [39].

### 2.2. Convolution Neural Networks

Convolutional Neural Network (CNN) is, now, the go-to method for pattern classification and image processing [40] and it has been proven to perform better than the traditional methods [41]. Although numerous unsupervised and recurrent variants have been developed, a CNN is a supervised feedforward artificial neural network. Literature has also demonstrated the importance of CNNs and its use in computer vision systems [2]. The visual cortex of the eye inspired the arrangement of neurons in CNNs [42]. Convolutional layers, pooling Layers, and nonlinear and fully connected (FC) layers are the components of CNN architecture [43].

The ImageNet Large Scale Visual Recognition Challenge (ILSVRC) has seen an increase in several CNNs and deep learning techniques commonly called pretrained networks. The AlexNet model developed in 2012 brought about other advanced architectures such as VGG, Xception, ResNet, and DenseNet which have been among performing techniques in recent times [44]. Pretrained networks used in this study are presented in the next section.

#### 2.2.1. Alexnet

AlexNet, considered largely as the first deep CNN architecture for classification and recognition tasks, has five convolution layers and three fully connected layers. A Local Response Normalization (LRN) was first introduced in AlexNet architecture with the ReLU activation function. AlexNet has a deeper architecture than its predecessor LeNet, which consists of five convolution layers, one pooling layer (max), a ReLU activation function, and three fully connected layers. It also used the dropout technique as its regularization method. [45].

#### 2.2.2. Googlenet

Three convolution layers, nine inception modules, with two levels each, and one fully-connected layer make up GoogleNet's 22-layer network. An inception block is used in the first layer of the GoogleNet architecture, which uses filters of 1 × 1, 3 × 3, and the 5 × 5 sizes. To improve performance, errors are calculated at numerous intermediate stages. A 1 × 1 concatenation filter is placed between them to alter input computations before moving on to the following layers' convolution kernels for processing. The number of features in the last layer is 1,024. To decrease the parameters numbers, the fully connected layers were replaced with a pooling layer. This reduced the parameters from 138 million to 4 million [46].

#### 2.2.3. Inceptionv3

An improved version of GoogleNet was introduced in 2015 which is the Inceptionv3. To improve performance, *n* × 1 and 1 × n convolutional layer factors are used instead of *n* x *n* factors. Filters with sizes of 5 × 5 were replaced with two of 3 × 3 filters in the architecture, which lead to a significant number of neurons reduction and parameters to 24 million. In this architecture, the convolutions are factorised into smaller convolutions [47].

#### 2.2.4. Vggnet

In 2014, Oxford University researchers introduced Visual Geometry Group (VGG) which differs slightly from AlexNet in terms of the kernel size and the number of feature maps. VGG architecture has thirteen convolutional layers with a max-pooling layer and three fully-connected layers following it. Large filters were replaced with smaller ones because developers believed running filters with lesser sizes concurrently could perform the same task. The VGG-16 and VGG-19 increased the layers numbers in the network as indicated by the numbers. The VGG-16 and VGG-19 architectures are made up of successive 3 × 3 convolution layers after which a pooling layer follows. The increase in the depth of the layer increased trainable parameters [48].

#### 2.2.5. Resnet

The theory behind designing the Residual Neural Network (ResNet) transformed the generation of CNN in 2015. It introduced the concept that higher layers learn new features from the previous layers. Connections added to layers are copied to the next layer's input without considering the extraction of features and identity from the previous layer. Even though it is having 152 layers which is 20 times more than AlexNet and 10 times more than VGG, it has a lower computational complexity than the other networks. The ResNet indicated a 3.57% error after training and implementation on the ImageNet dataset, which, as compared to the human error, is less [49].

#### 2.2.6. Mobilenet

One drawback of deep learning is that it is very expensive to train therefore Google researchers in 2017 introduced MobileNet to solve this resource constraint problem. In MobileNet which is a small low-consumption model, a normal convolutional layer was used instead of a deep convolutional layer. Deep convolutional layers process individually on each colour channel making it computationally intensive. MobileNet consists of 28 layers which in the absence of computing power become more appropriate for mobile-based vision programs. To improve training performance, depthwise convolution layers are being replaced with convolution layers [50].

#### 2.2.7. Densenet

DenseNet is a powerful neural network for image recognition that was first introduced in 2017. In DenseNet, features are rather transferred to all subsequent layers from all previous layers, as opposed to ResNet, which keeps each layer's information within the layers without offering to the next layer. This leaves subsequent layers with insufficient data and information to train. However, in a DenseNet, each subsequent layer in a dense block receives maps of the previous feature after which it is obtusely connected to each preceding layer, potentially reducing gradient calculations, decreasing the parameters numbers, and allowing features to be reused [51]. The core of the ResNet model is to train deeper CNNs by establishing shortcuts (skip connections) between the front and back layers, which helps to backpropagate the gradient during training. The DenseNet model is developed based on the same basic idea as ResNet, but it establishes dense connections between all of the previous and subsequent layers, which is reflected in its name. These features allow DenseNet to achieve better performance than ResNet with fewer parameters and less computational cost [52].

#### 2.2.8. LOG Gabor Filters

David Field proposed the Log-Gabor function [53]. Log-Gabor filters contain transfer functions that are consistent with the human visual system, which exhibits symmetric cell responses on the log frequency scale. Furthermore, observations on mammals' visual systems show that we have symmetric responses of cells on the log frequency scale, similar to the Log-Gabor function. Because the Log-Gabor transform has an extended tail with no DC component, it allows for the construction of infinitely broad bandwidth filters, which are able to encode natural images more effectively by expressing the higher frequency components.

When calculated on a logarithmic frequency scale, log-Gabor functions have Gaussian transfer functions. The 2D Log-Gabor filter is generated in the frequency domain because the log function at the origin has a singularity. The radial and angular filters make up the Log-Gabor function in polar coordinates. The response frequency of the radial filter is illustrated by the following equation [54]:

(1) $$\begin{array}{c} G_{r}\left(r\right)=\exp\;\left(-\frac{{\left[\log\;\left(r/f_{0}\right)\right]}^{2}}{2.\sigma_{r}^{2}}\right). \end{array}$$

And, the frequency response of the angular filter described by the following equation:

(2) $$\begin{array}{c} G_{\theta }\left(\theta \right)=\exp\left(-\frac{{\left[\theta -\theta_{0}\right]}^{2}}{2.\sigma_{\theta }^{2}}\right). \end{array}$$

#### 2.2.9. Related Works

Many researchers have used handcrafted features with supervised classifiers and deep learning models, particularly, CNN for plant identification.

Kan et al. [55] introduced an automatic system that uses the leaf for the classification of medicinal plants. The dataset contained 240 leaves of different plants from the medicinal plant specimen library of Anhui University of Traditional Chinese medicine. Five texture and ten shape features were extracted and using the SVM classifier and a 93.3 percent recognition rate was achieved. Begue et al. [56] extracted several leaf features such as a number of vertices, length, width, perimeter, and area of hull and colour on a dataset of 24 different plant species having 30 images each from the tropical island of Mauritius. The highest accuracy achieved was 90.1% using the random forest classifier.

De Luna et al. [57] experimented with seven algorithms (logistic regression, naïve bayes, K-Nearest Neighbor (KNN), linear discriminant analysis, classification and regression trees, SVM, and Neural Networks (NN) in identifying Philippine herbal medicine plants using leaf features. Various leaf shape and venation structure features were extracted and resulted in a 98.6% recognition rate. Vijayshree and Gopal [58] introduced a system using neural networks to classify and identify herbal medicinal plants on a dataset containing 50 different species having 500 leaves. A total of 21 features were extracted using texture, colour, and shape. Experimental results gave 93.3% accuracy using only texture features and 99.2% using all three features. Dahigaonkar and Kalyane [59] identified ayurvedic medicinal plants using leaf based on its colour texture and shape feature using SVM on 32 different plants. Features extracted include entropy, solidity, eccentricity, contrast, extent, standard deviation, mean, and equivalent diameter. An accuracy of 96.66% was achieved.

Britto and Pacifico [60] compared the performance of the Extreme Learning Machine (ELM) algorithm with K-Nearest Neighbor, Decision Tree classifier, Support Vector Machine, Naive Bayes classifier, and a Multilayer Perceptron trained with Backpropagation algorithm in the context of plant classification. The datasets, Fisher's Iris Plant, Wheat Seed Kernels, and 100 Plant Leaves were used in this investigation. A Centroid Contour Curve form signature, a fine-scale margin feature histogram, and an interior texture feature histogram were among the characteristics extracted. ELM achieved the best performances with the Iris data set (97%) and Seed data set (96%). The texture was presented as the best individual discriminatory power.

Dissanayake and Kumara [61] performed a comparison of the performance of multiple machine learning algorithms to identify herbal, fruit, and vegetable plants using their leaves. A total of 3,150 leaf photos from 25 different herbal, fruit, and vegetable species were used. Color photos were transformed to grayscale images, and the image noise was reduced using a Gaussian filter. Shape, texture, and colour are the three feature categories from which 17 features were collected. Support Vector Machine, K-Nearest Neighbors, Multilayer Perceptron, Random Forest, and Decision Tree algorithms have classification accuracy of 85.82 percent, 75.45 percent, 82.88 percent, 80.85 percent, and 64.39 percent, respectively.

Naeem et al. [62] developed a machine learning (ML) based medical plant leaf classification utilizing multispectral and texture datasets. A total of six varieties of medicinal plant leaves are used. Out of 65, 14 features were selected using a chi-square feature selection strategy. Five machine learning classifiers were used (multilayer perceptron, random forest, logit-boost, basic logistic, and bagging), with the multilayer perceptron classifier showing the most promise at 99.01 percent accuracy.

Xue et al. [63] showed that an ANN model developed using the morpho-colourimetric parameters as inputs performed better (accuracy of 98.3%) than a visible (VIS)/Near Infrared (NIR) spectral analysis (92.5% accuracy) when tested on 20 different Chinese medicinal plants which were sampled for their leaves. Kaur and Kaur [64] using the Swedish Leaf dataset applied the Gaussian filtering mechanism as a preprocessing technique after which texture and colour features were extracted. Classification using a Multiclass-support vector machine achieved an accuracy of nearly 93.26%. Singh [65] proposed the Local Binary Patterns—Support Vector Machine (LBP-SVM) methodology on the Swedish Leaf and compared it with the K-NN classifier and Binarized Neural Network (BNN). The LBP-SVM model provided a higher accuracy outcome of 84% while the existing BNN, and KNN models produced only 77% and 75%, respectively.

Nguyen et al. [66] used a pretrained network GoogLeNet to extract a 1024-dimensional feature vector of the last average pooling layer before the dropout layer. Ten classifiers are applied (Nearest neighbour (NNB), Linear Support Vector Machine (L_SVM), Nonlinear Support Vector Machine, Ada Boost (AB), Decision Tree (DT), Naïve Bayes (NB), Neural network (NN), Random Forest (RF), Quadratic discriminant analysis (QDA), and Softmax (SM), the default classification method of the GoogLeNet. The Linear SVM classifier achieved the best result of 87.34%. Jaiganesh et al. [67] proposed a Convolution Neural Network, which consists of four layers, convolution layer, dropout, max pooling, and average pooling. The proposed model was implemented using the Kaggle tool. The accuracy of the model was 86% which was accomplished with much less computational effort and shows the efficiency of the algorithm. Huynh et al. [68] suggested a five-layered Convolutional Neural Network (CNN) architecture in which the red channel of colors replaces the leaf vein shape data. To enhance the amount of training images, data augmentation was used to make three duplicates of each image after reflection and rotation. On the Flavia leaf data set and the Swedish leaf data, experimental findings showed that the suggested CNN model was successful for leaf recognition, with the greatest accuracy of greater than 98.22%.

Banzi and Abayo [69] proposed a CNN-LSTM network with 20 layers: 12 convolutional layers, one Fully Connected layer, five pooling layers, one Long Short-Term Memory (LSTM) layer, and one output layer with the softmax function for classification. Training of the models was performed by using an open database of 100 plant species images, containing 64 different element vectors of plants in a set of 100 distinct classes of plant species. Experiments showed that the proposed CNN-LSTM performs better in classifying plant species than the convectional CNN as it attains an accuracy of 95.06%. Karahan and Nabiyev [70] used a pretrained network MobileNetV2 to develop a plant identification system. A dataset containing 5,345 flowers and plant images belonging to 76 species was used. Preprocessing techniques used were centre cropping and normalizing. To expand the collection of images in the database and boost the model's generalization power, data augmentation techniques were also used. The suggested model attained a training set accuracy of 0.9971 and a test set accuracy of 0.9897 after 15 epochs. Pravin and Deepa [71] reviewed three different convolutional neural network algorithms; Matrix-Based Convolutional Neural Network (M-bCNN), Dual-path CNN (DP-CNN), and Fine-tuned AlexNet model for the medicinal leaf identification. DP-CNN produced the highest accuracy of 95.67%, Fine-tuned AlexNet model had 93.31%, and Dual-path CNN, 91%.

Chung et al. [72] created a dual-path CNN model in which the two subnetworks are independent and receive individual input from either an original image or a centrally cropped image. The suggested model attained a 77.1 percent accuracy rate after training and validation on a plant dataset of 14 species of Taiwan's most prevalent trees. Adetiba et al. [73] used the Leafsnap image dataset of 185 plant species and five pretrained CNN models (AlexNet, GoogLeNet, VGG-19, ResNet50, and MobileNetV2) to produce an accurate plant species recognition. MobileNetV2 with ADAM optimizer has the greatest testing accuracy of 92.3% among the pretrained models.

Bao et al. [74] devised a system for recognizing plants based on their leaf patterns that use two methods: a Histogram of Oriented Gradient (HoG) and a deep convolutional neural network. HoG was used to classify the features, while CNN was utilized to identify them. Ghazi et al. [75] implemented three models of transfer learning to explain better the identity of the various plants. These three-model used were GoogleNet, VGGNet, and AlexNet applied on the LIFECLEF 2015 dataset. The overall accuracy was 80% on the validation set and an overall inverse rank score of 0.752 on the official test set was achieved with the best-combined model. Krause et al. [76] presented a What's That Plant (WTPlant) system for identifying plants in natural images using deep learning approaches. Preprocessing was done using the Watershed Transform and the GrabCut and the classification engines used are two AlexNets pretrained models. In preliminary tests, the WTPlant system detected 99.3 percent of plants in 17,000 natural photos.

Sulc and Matas [77] combined the ResNet152 and Inception-ResNetv2 architectures with LBP and achieved an accuracy of 99% on the Swedish Leaf dataset. Zhang et al. [78] proposed a seven-layer CNN to classify the Flavia dataset and reached 94% accuracy. Pawara et al. [79] fine-tuned the AlexNet and GoogLeNet architectures and achieved 94% accuracy on Flavia, 98% on Folio and 99% on the Swedish Leaf dataset. Barre et al. [80] used a 17-layer CNN architecture and obtained an accuracy of 97.9% validated on the LeafSnap, Flavia, and Foliage datasets. [79] fine-tuned the AlexNet and GoogLeNet architectures and achieved 94% accuracy on Flavia, 98% on Folio and 99% on the Swedish Leaf dataset.

Pearline et al. [81] utilized VGG19 architecture with a logistic regression classifier on the Folio, Flavia, and Swedish leaf datasets, achieving an accuracy of 96%, 96%, and 99%, respectively. Blesslin and Baulkani [82] developed a proposed network AousethNet by replacing the SoftMax classifier with supervised learning classifiers; support vector machine (SVM), Decision tree (DT), Naive Bayes (NB) and K Nearest Neighbor (KNN), Ensemble classifier (EC) and the Majority vote classifier (MVC). With the Mendeley dataset (MD2020), the proposed model gave an accuracy of 99% and a precision of 98% with the MVC.

A summary of the reviewed papers is presented in Table 1 and Table 2.

## 3. Methodology

### 3.1. Dataset

A medicinal plant leaf dataset (MyDataset) [83] has been developed for this study from the Centre for Plant Medicine Research (CPMR) in Akuapem Akropong, Ghana and is presented in Table 3. The digital images are acquired using the NIKON D3500 camera on the abaxial portions of the medicinal plant leaves, in the uncompressed JPEG format in YCbCr colour format with the dimension 6000 × 4000 × 3, in a closed environment to maintain constant illumination. The benchmark standards followed to create the dataset includes relevance, representativeness, nonredundancy, experimentally verified cases, scalability, and reusability [84, 85]. The working dataset consists of 2450 images, 50 images each from 49 medicinal plant leaves. The dataset created (MyDataset) is compared with four benchmark datasets which are: Flavia dataset, Swedish Leaf dataset, Mendeley Dataset (MD2020), and Folio dataset. Flavia dataset contains 1907 samples of 33 species of common native plants in Yangtze Delta, China [86, 87]. All the leaf images in this dataset have no petioles. The Swedish dataset contains 75 samples from each of the 15 Species of Swedish plants or trees [88, 89]. The Folio dataset contains 576 images with 18 samples each from 32 species [90] whiles the Mendeley dataset contains 1835 images from 30 species [91].

### 3.2. Feature Extraction

Pretrained CNN networks which includes AlexNet, inceptionv3, DenseNet201, GoogLeNet, resnet101, resnet18, resnet50, mobilenetv2, vgg16, and vgg19 are used as feature extractors. The process of using features from a pretrained network is known as transfer learning [92]. Transfer learning aims to increase target learners' performance on target domains by transferring data from a variety of related source domains. The dependency on a big amount of target domain data for developing target learners can be decreased using this strategy. The last layer before the fully connected layer for classification commonly called the bottleneck layers are used in this study. A description of the pretrained network and the layers used are presented in Table 4.

### 3.3. Supervised Classifiers

Six (6) well-known supervised learning algorithms are chosen for this study. These classifiers are chosen based on the following groups: Bayesian, lazy classifiers, trees, and functions [93]. Bayesian classifiers assign membership probability to new objects in order to categorize them, and they are known to be quick and accurate even when dealing with massive amounts of data [94]. From a training set of objects represented by various qualities, trees derive rules. Because the derived rules may be represented as a treelike graph, they can be easily understood [95]. Lazy (or instance-based) classifiers save all of the training samples and wait until a new instance needs to be classified before building a classifier. During the training phase, lazy-learning algorithms require less calculation time, but during the classification phase, they require greater computation time [96]. Functions or nonprobabilistic classifiers in this category strive to generalize the training data before accepting queries. The majority of the methods in this family can be thought of as simple applications of optimization theory and statistical estimation [97]. The classifiers used are Naive Bayes, Support Vector Machine (SVM), K-nearest Neighbour (kNN), Decision Tree (DT), Logistic Regression (LR), and Random Forest (RF).

### 3.4. Proposed Model

**Figure 1:**
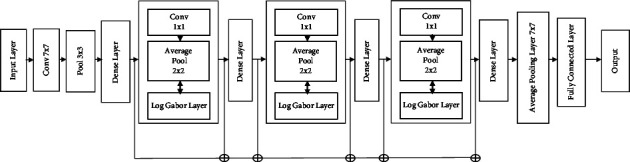
Proposed CNN model.

The proposed CNN model (OTAMNet) created is based on the DenseNet architecture since it has the following advantages: since error signals can be easily transferred to older levels more directly, there is a significant gradient flow; an implicit deep supervision is provided because the final classification layer provide strict supervision to earlier levels; more diversified features can be extracted because each layer in DenseNet receives all preceding layers as input as opposed to standard CNN models where the classifier uses the most complex features [98]. In DenseNet, the classifier uses features of all complexity levels, giving more smooth decision boundaries [98]. The model fused handcrafted features i.e., Log-Gabor features into each dense block of the DenseNet model and propagated features to each next dense block as shown in Figure 1. The minimum and maximum frequencies, the filter bandwidth to employ, the scaling between center frequencies of subsequent filters, the number of orientations, the number of scales, and the angular spread of each filter are the parameters used in designing a Log-Gabor filter [99]. The parameters used are presented in Table 5 [100].

Features from each dense block are fused with the log Gabor features before being sent to the next block allowing for rich complex features at each stage.

### 3.5. Classification Metrics

The confusion matrix is the most often used assessment measure in predictive analysis, owing to its simplicity and ability to compute other important metrics including accuracy, recall, and precision. It is an NxN matrix that depicts a model's overall performance when applied to a dataset, where N represents the number of class labels in the classification task. These metrics are as follows [101]:

True Positive (TP) refers to a situation in which the actual value was positive and the predicted value was positive as well
False Positive (FP) refers to a situation in which the actual value is negative but the predicted value is positive
True Negative (TN) refers to a situation in which the actual value was negative and the predicted value was negative as well
False Negative (FN) refers to a situation in which the actual value is positive but the predicted value is negative
Accuracy (ACC) is the fraction of appropriately classified connections (true positives and true negatives) over the total number of connections in the dataset
  (3) $$\begin{array}{c} ACC=\frac{TP+TN}{N}, \end{array}$$
 where *N* = number of instances. The fraction of true positives to actual positive cases is known as Recall, also known as sensitivity or True Positive Rate (TPR). Simply said, recall is the number of true positives discovered (recalled) out of all true positive cases.
  (4) $$\begin{array}{c} TPR=\frac{TP}{TP+FN}. \end{array}$$
Precision, also known as Positive Predictive Value (PPV), is the ratio of true positives to false positives. Simply put, precision refers to how many of the cases discovered were true positives.
  (5) $$\begin{array}{c} PPV=\frac{TP}{TP+FP}. \end{array}$$
False Positive Rate (FPR) or “Fall-Out”: this is the percentage of negative cases in the data that are mistakenly recognized as positive (i.e., the probability that false alerts will be raised).
  (6) $$\begin{array}{c} FPR=\frac{FP}{FP+TN}. \end{array}$$
The harmonic mean of recall and precision is the F1 score, often known as the F score or F-measure. Its value varies from 0 to 1, with 0 being the worst and 1 being the greatest. It can be calculated in the following way.
  (7) $$\begin{array}{c} F_{1}=\frac{2*\left(PPV*TPR\right)}{PPV+TPR}. \end{array}$$

## 4. Analysis

**Figure 2:**
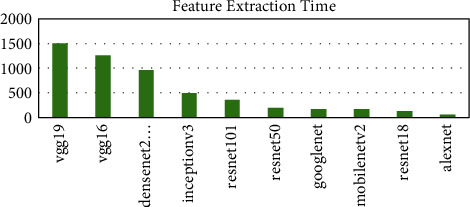
Feature Extraction Time for Pretrained networks.

**Figure 3:**
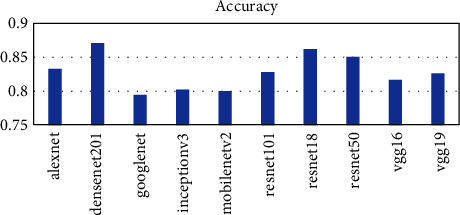
Accuracy metric for Pretrained networks.

**Figure 4:**
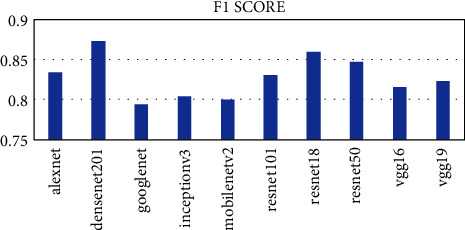
F1 score metric for Pretrained networks.

**Figure 5:**
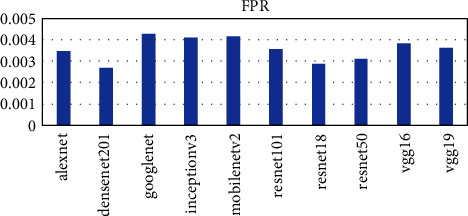
FPR score metric for Pretrained networks.

**Figure 6:**
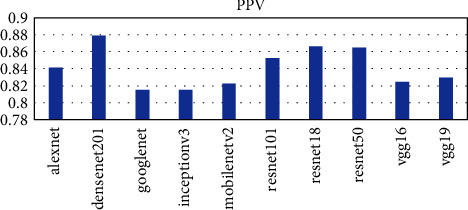
PPV score metric for Pretrained networks.

**Figure 7:**
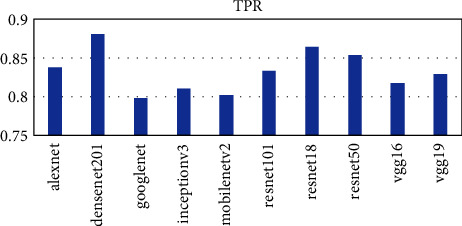
TPR score metric for Pretrained networks.

Ten pretrained networks were used to extract complex features from the leaf images. The number of features extracted is directly connected to the layer at which the feature was extracted. The layers used in each pretrained network are the last layer before the fully connected layer for classification. The number of features extracted ranges from 512 to 4096. The AlexNet, vgg16, and vgg19 extracted the highest number of features of 4096 whiles the resnet18 extracted the lowest number of features of 512. The time taken for each pretrained network is not directly proportional to the number of features but the complexity of the pretrained network. From Figure 2, it can be seen that the vgg19 performed worse in terms of time taken and AlexNet performed the best taking a time of about 54 seconds to complete as compared to 1498 seconds for vgg19. The DenseNet201 model also used about 956 seconds to complete which was the third worse performance in terms of time. To ascertain why the DenseNet201 model was chosen as the base model for OTAMNet, the features derived from the pretrained network were used for the classification process using the six supervised learning algorithms mentioned earlier. The average result from the six classifiers was used as a benchmark to check the performance across the various metrics. The results are presented in Figures 3–7.

As shown in Figure 3 to Figure 7, the DenseNet201 performed the best in terms of accuracy, F1 score, False Positive Rate, True Positive Rate, and Positive Predictive Value. In terms of accuracy, DenseNet201 scored 87% with resnet50 following with 85% and GoogLeNet performing the worse with 79%. With the F1 score, DenseNet201 also performed the best with 87%, resnet18 followed with 86% and GoogLeNet scored the lowest with 79%. DenseNet201 also had the lowest False Positive Rate with 0.26%, resnet18 following with 0.28% and the highest being 0.42% by GoogLeNet. DenseNet201 outperformed all other networks also in terms of the True Positive Rate and Positive Predictive Value scoring 88% and 87% respectively. The resnet50, resnet18, and resnet101 follow with 86%, 85%, and 83%, respectively for the True Positive rate and 86%, 86%, and 85% in terms of the Positive Predictive Value.

**Figure 8:**
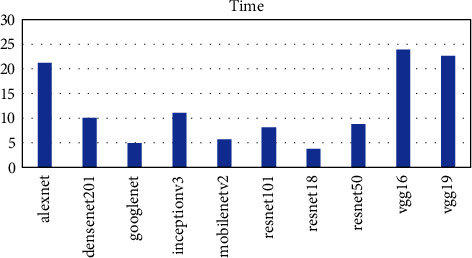
Time metric for Pretrained networks.

The time taken for the classifier to complete its operations was also taken into consideration as presented in Figure 8 vgg19, vgg16, and AlexNet had the highest running time of 23, 22, and 21 seconds as compared to resnet18, GoogLeNet, and mobilenetv2 having the lowest time of 3, 4, and 5 seconds, respectively. DenseNet201 performed fairly well with 10 seconds as shown in Figure 8. Overall, the denset201 model proved to be the best pretrained model to be used as the base model for developing OTAMNet.

OTAMNet was implemented in the python environment and tested on the dataset “MyDataset” created by the researcher and four benchmark datasets. The metrics used during the training of the model are accuracy and loss. A 70 : 30 ratio was used for splitting the dataset into training and testing sets. The model was also validated using a 33% validation split on the training data. The Adam optimizer was used during the training of the model. For training the model, the Google colab server was used to boost the running time of the model. To check for overfitting, an EarlyStopping callback was used to check the minimum validation loss with a waiting time of 5 epochs to stooped the model if the validation loss starts to increase after the waiting period. Early stopping is a method of training that allows a set of an arbitrary large number of training epochs and then terminate when the improvement of the model's performance on the validation dataset stops. A ModelCheckpoint was also used to only save and use the best weights which are derived from the model. To reduce the running time and improve performance, a batch size of 8 was used for both training and testing of the model.

**Figure 9:**
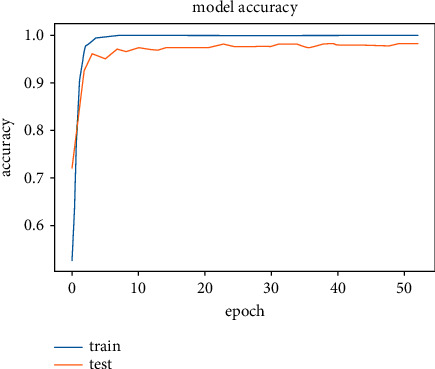
Model accuracy for MyDataset.

**Figure 10:**
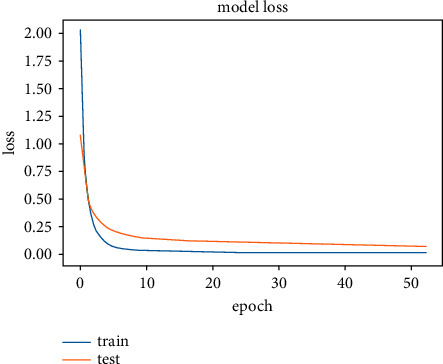
Model loss for MyDataset.

The results obtained after running the OTAMNet model on MyDataset is presented in Figure 9 and 10.

An accuracy metric is used to calculate the algorithm's processing ability in an interpretable way. It is a metric that measures how closely your model's predictions match the actual facts. A loss function is used to fine-tune a machine-learning algorithm. Training and validation are used to estimate the loss, and the model's performance in these two sets determines the significance of the loss. The sum of all errors made during each training or validation set is calculated for each example. After each optimization cycle, the loss value shows how well or poorly a model performs. The training accuracy reached 100% and the training loss also decreased from 2 to 0.2 after the 9^th^ epoch. The validation accuracy increased from the 1^st^ to the 5^th^ epoch, then the 7^th^, 8^th^,15^th^, 35^th^, and 51st epoch. The EarlyStopping model kicked in at the 53rd epoch and the training stopped because the model performance stopped improving on the validation dataset. The validation accuracy for the model was 98% with a loss of 0.08.

The model was saved and used to predict a new set of data and the results are presented in Table 6.

75% of the Plant leaf tested had 100% accuracy and 25% had a 99% accuracy. OTAMNet also performed well in terms of the False Positive Rate with 87% of the dataset scoring 0% with the highest FPR being 0.04%. 87% of the dataset had a TPR of 1 showing a high rate of predicting positive classes.

**Figure 11:**
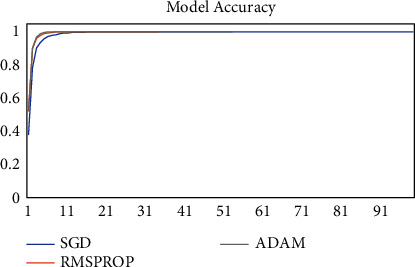
Model accuracy on optimizers.

**Figure 12:**
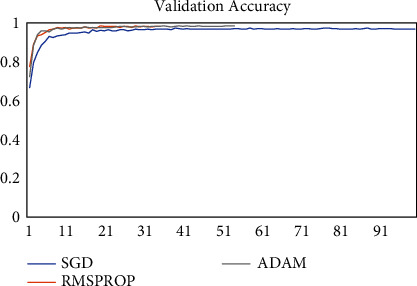
Validation accuracy on optimizers.

**Figure 13:**
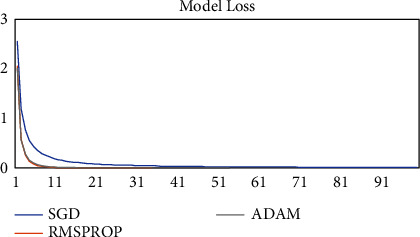
Model loss on optimizers.

**Figure 14:**
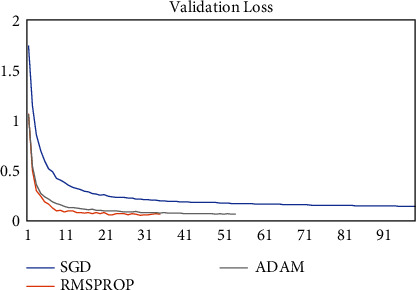
Validation loss on optimizers.

The model was trained on two other optimizers, Stochastic Gradient Descent (SGD) and the Root Mean Square Propagation (RMSProp) to determine its performance which is presented in Figures 11–14.

It could be seen that the Adam and RMSProp optimizer both had a sharp curve after the 10^th^ epoch as compared to the SGD. Overfitting occurred earlier in the RMSProp optimizer i.e., at the 34^th^ epoch as compared to the Adam optimizer which occurred at the 53^rd^ epoch whiles the SGD run on the maximum epoch allocated which was 100. When the model was tested on the test data, the Adam optimizer produced the highest accuracy of 98% and the lowest loss of 0.08 as compared to the SGD and RMSProp. This is presented in Table 7 and a summary of the analysis is presented in Table 8 using the Adam optimizer.

**Figure 15:**
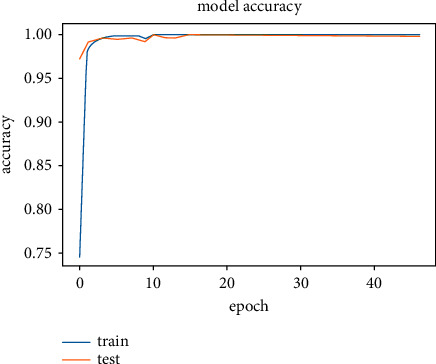
Model accuracy for flavia dataset.

**Figure 16:**
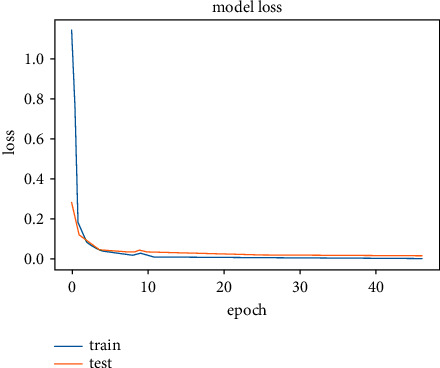
Model loss for flavia dataset.

OTAMNet was also tested on the Flavia dataset having 32 plant species. Early Stopping kicked in at the 47^th^ epoch to prevent overfitting of the data. The model achieved 99% accuracy on the validation data set and a loss of 0.01 as shown in Figures 15 and 16.

The model, when used on the test data had an accuracy of 99.4%, F1 score of 99.4%, TPR of 99.4%, PPV of 99.5% and an FPR of 0.00017, and the full metrics are presented in Table 9.

**Figure 17:**
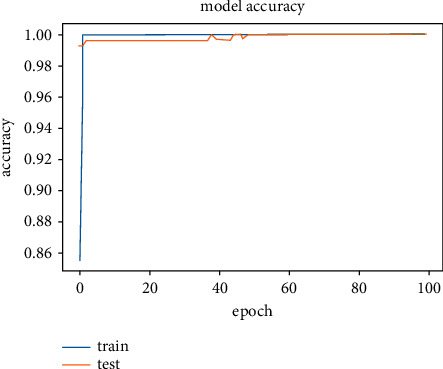
Model accuracy for Swedish leaf dataset.

**Figure 18:**
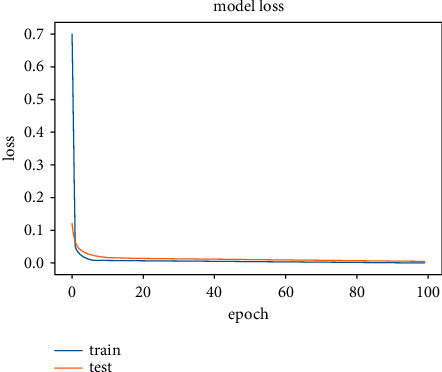
Model loss for Swedish leaf dataset.

The Swedish data produce a 100% accuracy with a loss of 0.00 both on the training and validation test data. The model also predicted correctly all test data samples and this is presented in Figures 17 and 18 and Table 10.

**Figure 19:**
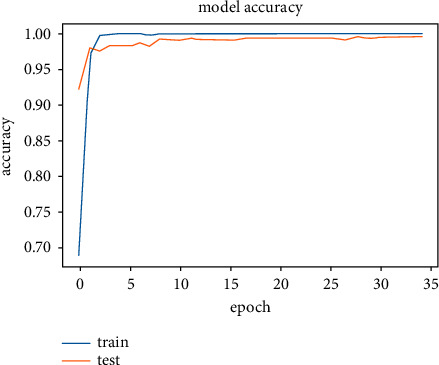
Model accuracy for mendeley dataset.

**Figure 20:**
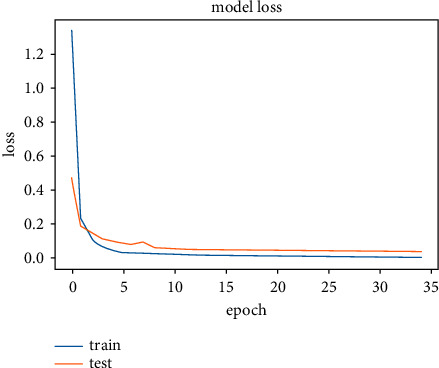
Model loss for mendeley dataset.

When tested on the Mendeley dataset having 30 plant species, OTAMNet achieved 99% accuracy on the validation data set and a loss of 0.06. Early Stopping kicked in at the 35^th^ epoch to prevent overfitting the data as shown in Figures 19 and 20. The model when the run of the test data had an accuracy of 98.6%, F1 score of 98.4%, TPR of 98.3%, PPV of 98.6%, and an FPR of 0.00048. The performance metric for each leaf in the dataset is presented in Table 11.

**Figure 21:**
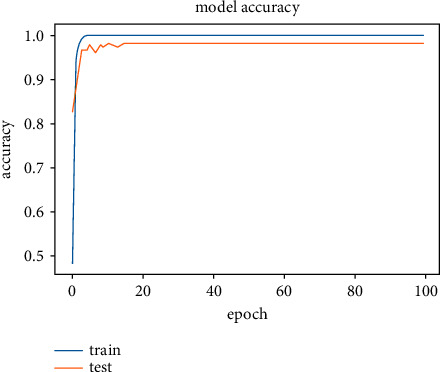
Model accuracy for folio dataset.

**Figure 22:**
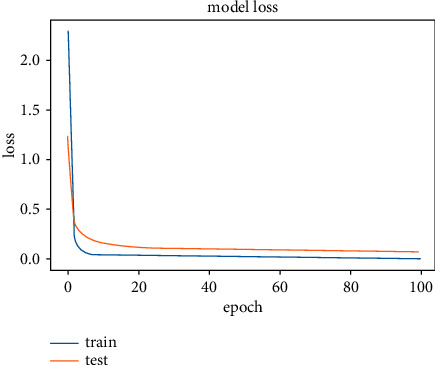
Model loss for mendeley dataset.

OTAMNet achieved 97% accuracy on the validation data set and a loss of 0.08 when tested on the Folio dataset having 32 plant species as shown in Figures 21 and 22. The model when run on the test data had an accuracy of 96.8%, F1 score of 96.3%, TPR of 96.5%, PPV of 97.7%, and an FPR of 0.00104. The performance metric for each leaf in the Folio dataset is presented in Table 12.

**Figure 23:**
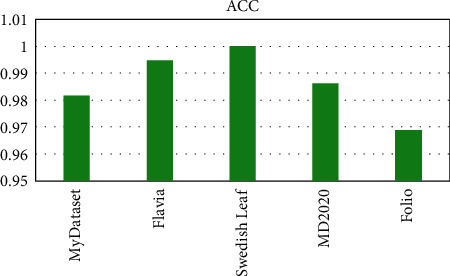
Model accuracy for all dataset.

**Figure 24:**
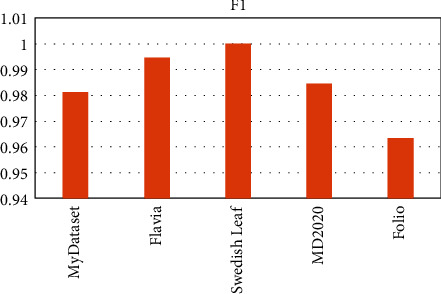
F1 score for all dataset.

**Figure 25:**
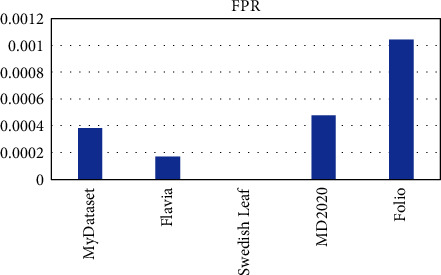
Fpr for all dataset.

**Figure 26:**
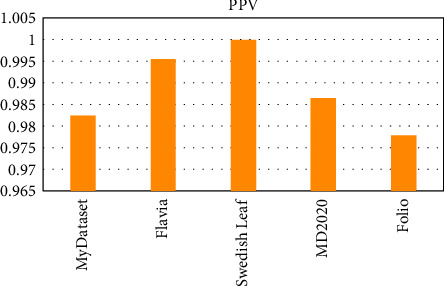
PPV for all dataset.

**Figure 27:**
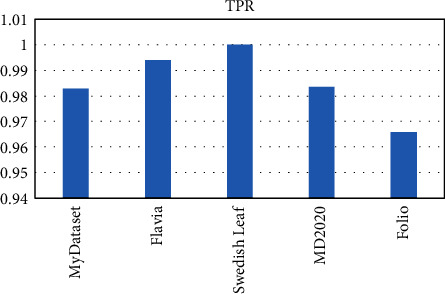
Tpr for all dataset.

**Figure 28:**
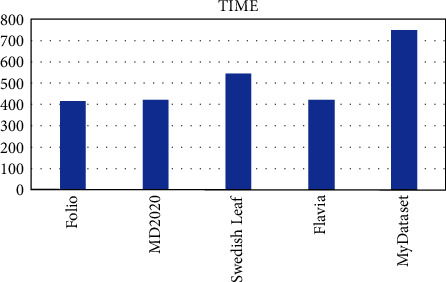
Running time for all dataset.

OTAMNet performed extremely well on all datasets used achieving an accuracy of 98%, 99%, 100%, 99%, and 98% on MyDataset, Flavia, Swedish Leaf Dataset, MD2020, and Folio dataset, respectively. The False Positive rate achieved was also insignificant with the highest being 0.000104 on the Folio dataset. A comparative summary of the results on all the datasets is presented in Figure 23–28.

The proposed system was compared with existing systems which have been reviewed in this study and the summary is presented in Table 13.

## 5. Conclusion

This study was carried out to identify plants based on their textural features using Log-Gabor filters and deep learning techniques. OTAMNet which was created by fusing a log Gabor layer into the transition layer of the DenseNet201 architecture achieved an accuracy of 98%. OTAMNet was tested on other benchmark datasets: the Flavia, Swedish Leaf, MD2020, and the Folio dataset. The Flavia dataset achieved 99%, Swedish Leaf 100%, MD2020 99%, and the Folio dataset 97%. A false-positive rate of less than 0.1% was achieved in all cases. For future works, the medicinal plant database can be enhanced to have more species across the nation and their variations depending upon climatic conditions so also their applications. The study can also be extended to classifying medicinal plants based on anatomical (studying of the internal structure of plants, which usually takes place at the microscopic/cellular level) and chemo-taxonomical (in classifying plants using their chemical constituents) properties.

## Figures and Tables

**Table 1:** Handcrafted features with supervised classifiers.

Reference	Features	Dataset	Algorithm	Accuracy (%)
[55]	Texture and shape features	Medicinal plant specimen library of anhui university of traditional Chinese medicine	SVM classifier	93.3%

[56]	Perimeter, a number of vertices, length, width, perimeter and area of hull, colour	Dataset of 24 different plant species having 30 images each from the tropical island of Mauritius	Random forest classifier	90.1%

[57]	Leaf shape and venation structure features	Philippine herbal medicine plants using leaf features	Logistic regression, naïve bayes, K-nearest neighbor (KNN), linear discriminant analysis, classification and regression trees, SVM, and neural networks (NN)	98.6%

[58]	Texture, colour, and shape	Herbal medicinal plants on a dataset containing 50 different species having 500 leaves.	Neural networks	93.3%

[59]	Color, texture and shape feature	Ayurvedic medicinal plant	SVM	96.66%

[60]	Centroid contour curve form signature, a fine-scale margin feature histogram and an interior texture feature histogram	Fisher's iris plant, wheat seed kernels, and 100 plant leaves	Extreme learning machine (ELM) algorithm with K-nearest neighbor, decision tree classifier, support vector machine, naive bayes classifier, and a multilayer perceptron trained with backpropagation algorithm	Iris data set (97%) Seed data set (96%).

[61]	Shape, texture, and colour	A total of 3,150 leaf photos from 25 different herbal, fruit, and vegetable species	Support vector machine, K-nearest neighbors, multilayer perceptron, random forest, and decision tree algorithms	85.82

[62]	14 features were selected using a chi-square feature selection strategy	Six varieties of medicinal plant leaves	Multilayer perceptron, random forest, logit-boost, basic logistic, and bagging	99.01%

[63]	Morpho-colourimetric parameters Visible (VIS)/Near infrared (NIR) spectral analysis	20 different Chinese medicinal plants	ANN model	98.3%

[64]	Texture and colour features	Swedish leaf dataset	Multiclass-support vector machine	93.26%.

**Table 2:** Deep learning models.

Reference	Algorithm	Dataset	Accuracy (%)
[66]	GoogLeNet + linear SVM		87.34%.
[67]	Convolution neural network		86%
[68]	Five-layered convolutional neural network (CNN)	Flavia leaf dataset	98.22%.
		Swedish leaf dataset	
[69]	CNN-LSTM network with 20 layers		95.06%.
[70]	MobileNetV2		98.97
[71]	Dual-path CNN (DP-CNN)		95.67%
[72]	Dual-path CNN model	14 species of Taiwan's most prevalent trees	77.1%
[73]	AlexNet, GoogLeNet, VGG-19, ResNet50, and MobileNetV2	Leafsnap image dataset	92.3%
[74]	5-Layer CNN architecture	Flavia leaf dataset Swedish leaf dataset	95.5 98.2
[75]	GoogleNet, VGGNet, and AlexNet	LIFECLEF 2015 dataset	80%
[76]	Two AlexNets pretrained models		99.3%
[77]	ResNet152 and Inception-ResNetv2 architectures with LBP	Swedish leaf dataset	99%
[78]	Seven-layer CNN	Flavia dataset	94%
[79]	AlexNet and GoogLeNet	Flavia	94% 98% 99%
		Folio	
		Swedish leaf dataset	
[80]	17-Layer CNN architecture		97.9%
[81]	VGG19 architecture with a logistic regression classifier	Folio	96% 96% 99%,
		Flavia	
		Swedish leaf datasets	
[82]	AousethNet	Mendeley dataset (MD2020	99%

**Table 3:** Description of dataset.

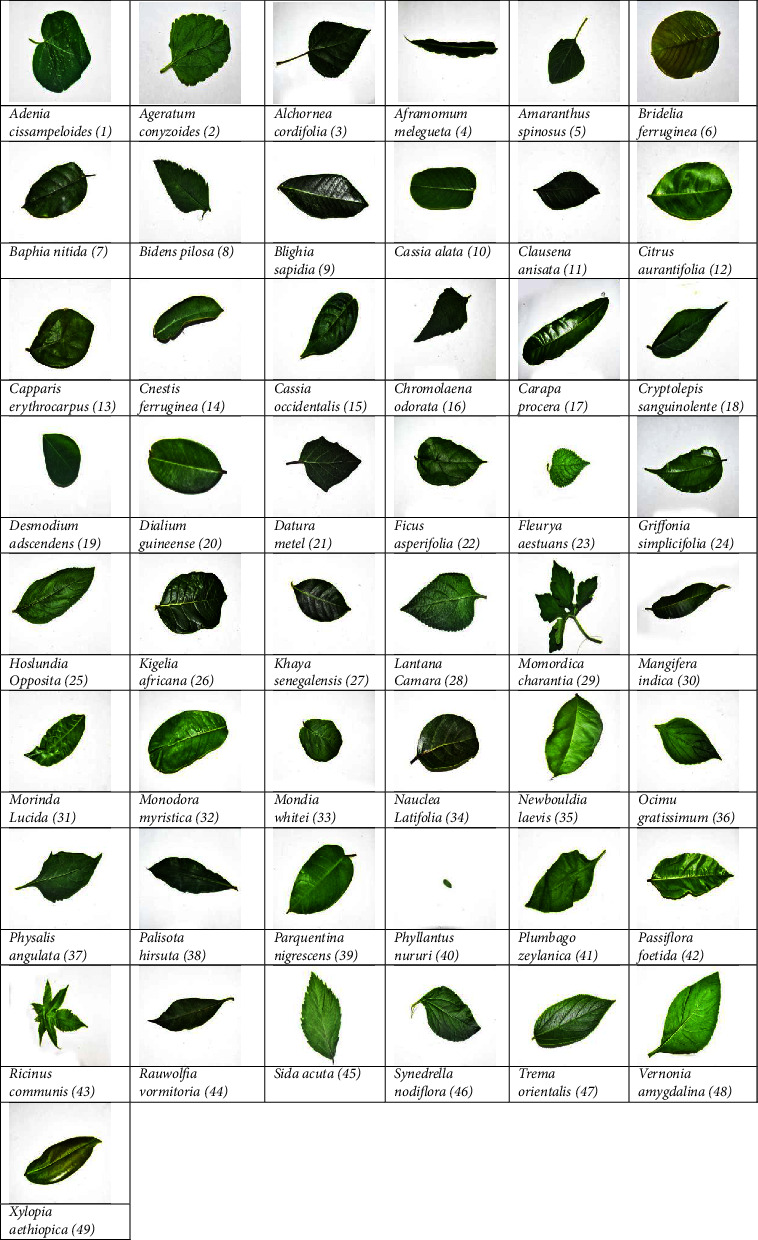

**Table 4:** Description of pretrained networks.

No	Network	Image input size	Depth	No of features	Layer
1	Alexnet	227-By-227	8	4096	fc7
2	DenseNet201	224-By-224	201	1920	avg_pool
3	Googlenet	224-By-224	22	1024	pool5-7x7_s1
4	inceptionv3	299-By-299	48	2048	avg_pool
5	mobilenetv2	224-By-224	53	1280	global_average_pooling2d_1
6	resnet18	224-By-224	18	512	pool5
7	resnet50	224-By-224	50	2048	avg_pool
8	resnet101	224-By-224	101	2048	pool5
9	vgg16	224-By-224	16	4096	fc7
10	vgg19	224-By-224	19	4096	fc7

**Table 5:** Log-gabor parameters.

Parameter	Value
Number of filter scales	8
Number of filter orientations	10
Minimum frequency	3
Scaling between centre frequencies	2
Filter bandwidth	0.65
Angular spread of each filter	1.5

**Table 6:** Metrics for mydataset.

Plant leaf	ACC	F1	TPR	FPR	PPV
1	1	1	1	0	1
2	0.99592	0.90909	1	0.00417	0.83333
3	0.99796	0.94118	1	0.00207	0.88889
4	1	1	1	0	1
5	0.99796	0.96552	1	0.0021	0.93333
6	1	1	1	0	1
7	1	1	1	0	1
8	1	1	1	0	1
9	1	1	1	0	1
10	1	1	1	0	1
11	1	1	1	0	1
12	1	1	1	0	1
13	0.99388	0.89655	0.8125	0	1
14	0.99796	0.90909	0.83333	0	1
15	1	1	1	0	1
16	1	1	1	0	1
17	1	1	1	0	1
18	1	1	1	0	1
19	1	1	1	0	1
20	0.99592	0.875	1	0.00414	0.77778
21	1	1	1	0	1
22	0.99796	0.92308	0.85714	0	1
23	1	1	1	0	1
24	1	1	1	0	1
25	1	1	1	0	1
26	1	1	1	0	1
27	1	1	1	0	1
28	1	1	1	0	1
29	1	1	1	0	1
30	1	1	1	0	1
31	1	1	1	0	1
32	0.99796	0.94118	1	0.00207	0.88889
33	0.99796	0.95652	0.91667	0	1
34	1	1	1	0	1
35	1	1	1	0	1
36	0.99592	0.9	1	0.00416	0.81818
37	1	1	1	0	1
38	0.99796	0.97143	0.94444	0	1
39	1	1	1	0	1
40	1	1	1	0	1
41	1	1	1	0	1
42	1	1	1	0	1
43	1	1	1	0	1
44	1	1	1	0	1
45	0.99592	0.88889	0.8	0	1
46	1	1	1	0	1
47	1	1	1	0	1
48	1	1	1	0	1
49	1	1	1	0	1

**Table 7:** Metrics based on optimizer.

Optimizer	Accuracy (%)	Loss
Adam	98	0.08
RMSProp	97	0.11
SGD	97	0.14

**Table 8:** Overall statistics.

Metric	Score
ACC	0.98163
F1	0.98117
FPR	0.00038
PPV	0.98246
TPR	0.98294

**Table 9:** Metrics for flavia dataset.

Plant leaf	ACC	F1	TPR	FPR	PPV
1	1	1	1	0	1
2	1	1	1	0	1
3	1	1	1	0	1
4	1	1	1	0	1
5	1	1	1	0	1
6	0.99738	0.9697	1	0.00273	0.94118
7	1	1	1	0	1
8	1	1	1	0	1
9	1	1	1	0	1
10	1	1	1	0	1
11	1	1	1	0	1
12	1	1	1	0	1
13	1	1	1	0	1
14	0.99738	0.93333	0.875	0	1
15	1	1	1	0	1
16	1	1	1	0	1
17	1	1	1	0	1
18	1	1	1	0	1
19	1	1	1	0	1
20	1	1	1	0	1
21	1	1	1	0	1
22	1	1	1	0	1
23	1	1	1	0	1
24	1	1	1	0	1
25	1	1	1	0	1
26	1	1	1	0	1
27	0.99738	0.95652	1	0.0027	0.91667
28	1	1	1	0	1
29	1	1	1	0	1
30	1	1	1	0	1
31	0.99738	0.96774	0.9375	0	1
32	1	1	1	0	1

**Table 10:** Metrics for swedish leaf dataset.

Plant leaf	ACC	F1	TPR	FPR	PPV
1	1	1	1	0	1
2	1	1	1	0	1
3	1	1	1	0	1
4	1	1	1	0	1
5	1	1	1	0	1
6	1	1	1	0	1
7	1	1	1	0	1
8	1	1	1	0	1
9	1	1	1	0	1
10	1	1	1	0	1
11	1	1	1	0	1
12	1	1	1	0	1
13	1	1	1	0	1
14	1	1	1	0	1
15	1	1	1	0	1

**Table 11:** Metrics for mendeley dataset.

Plant leaf	ACC	F1	TPR	FPR	PPV
1	1	1	1	0	1
2	1	1	1	0	1
3	1	1	1	0	1
4	1	1	1	0	1
5	0.99455	0.96154	1	0.00585	0.92593
6	1	1	1	0	1
7	1	1	1	0	1
8	1	1	1	0	1
9	0.99455	0.9	0.81818	0	1
10	1	1	1	0	1
11	1	1	1	0	1
12	1	1	1	0	1
13	1	1	1	0	1
14	1	1	1	0	1
15	0.99183	0.91429	0.88889	0.00287	0.94118
16	1	1	1	0	1
17	0.99728	0.96296	1	0.00282	0.92857
18	1	1	1	0	1
19	1	1	1	0	1
20	1	1	1	0	1
21	1	1	1	0	1
22	1	1	1	0	1
23	1	1	1	0	1
24	1	1	1	0	1
25	1	1	1	0	1
26	1	1	1	0	1
27	1	1	1	0	1
28	1	1	1	0	1
29	1	1	1	0	1
30	0.99455	0.8	0.8	0.00276	0.8

**Table 12:** Metrics for folio dataset.

Plant leaf	ACC	F1	TPR	FPR	PPV
1	1	1	1	0	1
2	1	1	1	0	1
3	0.98438	0.83333	1	0.01626	0.71429
4	1	1	1	0	1
5	1	1	1	0	1
6	1	1	1	0	1
7	0.99219	0.85714	0.75	0	1
8	1	1	1	0	1
9	1	1	1	0	1
10	1	1	1	0	1
11	1	1	1	0	1
12	1	1	1	0	1
13	1	1	1	0	1
14	1	1	1	0	1
15	1	1	1	0	1
16	0.98438	0.75	1	0.016	0.6
17	1	1	1	0	1
18	1	1	1	0	1
19	1	1	1	0	1
20	1	1	1	0	1
21	1	1	1	0	1
22	0.99219	0.92308	0.85714	0	1
23	1	1	1	0	1
24	1	1	1	0	1
25	1	1	1	0	1
26	0.98438	0.5	0.33333	0	1
27	1	1	1	0	1
28	1	1	1	0	1
29	1	1	1	0	1
30	1	1	1	0	1
31	1	1	1	0	1

**Table 13:** Comparison with existing systems.

Source	Method	Dataset	Accuracy (%)
OTAMNet	Log-gabor filter and DenseNet201	MyDataset	98 99 100 97 99
		Flavia	
		Swedish	
		Folio	
		MD2020	
[82]	Modified AlexNet	MD2020	99
[73]	AlexNet, GoogLeNet, VGG-19, ResNet50, and MobileNetV2	Leafsnap	92
[65]	Binarized Neural Network (BNN)	Swedish leaf	77
[68]	CNN	Flavia Swedish leaf	98
[74]	Histogram of oriented gradient (HoG) and deep convolutional neural network	Flavia Swedish leaf dataset	96
[81]	VGG19 with LR	Folio Flavia Swedish leaf dataset	96
			96
			99
[102]	AlexNet and VGG16 with LDA	Swedish leaf dataset	99
[80]	17-Layer CNN architecture	LeafSnap	97
		Flavia	
		Foliage datasets	
[79]	AlexNet and GoogLeNet	Flavia Swedish leaf dataset	94
			99
[75]	GoogLeNet, VGGNet, and AlexNet	LifeClef 2015 dataset	80
[103]	26-Layer CNN architecture	BJFU100 dataset	91
[78]	7-Layer CNN architecture	Flavia dataset	94
[77]	ResNet152 and Inception-ResNetv2 with LBP	Swedish leaf dataset	99

## Data Availability

The data used to support the findings of this study are included within the article.
